# Impact of Cytokines and Phosphoproteins in Response to Chronic Joint Infection

**DOI:** 10.3390/biology9070167

**Published:** 2020-07-16

**Authors:** Nicole Prince, Julia A. Penatzer, Matthew J. Dietz, Jonathan W. Boyd

**Affiliations:** 1 C. Eugene Bennett Department of Chemistry, West Virginia University, Morgantown, WV 26506, USA; aprince@mix.wvu.edu (N.P.); jamouch@mix.wvu.edu (J.A.P.); 2 Department of Orthopaedics, School of Medicine, West Virginia University, Morgantown, WV 26506, USA; mdietz@hsc.wvu.edu; 3 Department of Physiology and Pharmacology, West Virginia University, Morgantown, WV 26506, USA; 4 Department of Occupational and Environmental Health Sciences, West Virginia University, WV 26506, USA

**Keywords:** infection, inflammation, network analysis, PJI, cytokine, phosphoprotein

## Abstract

The early cellular response to infection has been investigated extensively, generating valuable information regarding the mediators of acute infection response. Various cytokines have been highlighted for their critical roles, and the actions of these cytokines are related to intracellular phosphorylation changes to promote infection resolution. However, the development of chronic infections has not been thoroughly investigated. While it is known that wound healing processes are disrupted, the interactions of cytokines and phosphoproteins that contribute to this dysregulation are not well understood. To investigate these relationships, this study used a network centrality approach to assess the impact of individual cytokines and phosphoproteins during chronic inflammation and infection. Tissues were taken from patients undergoing total knee arthroplasty (TKA) and total knee revision (TKR) procedures across two tissue depths to understand which proteins are contributing most to the dysregulation observed at the joint. Notably, p-c-Jun, p-CREB, p-BAD, IL-10, IL-12p70, IL-13, and IFN-γ contributed highly to the network of proteins involved in aseptic inflammation caused by implants. Similarly, p-PTEN, IL-4, IL-10, IL-13, IFN-γ, and TNF-α appear to be central to signaling disruptions observed in septic joints. Ultimately, the network centrality approach provided insight into the altered tissue responses observed in chronic inflammation and infection.

## 1. Introduction

Acute responses to inflammation and infection have been well studied in literature, and these studies have highlighted important roles for many cytokines [1,2,3] and phosphoproteins [4,5] in early inflammatory immune processes. The coordinated series of signaling events involves the recruitment of pro-inflammatory regulators like IL-1α, IL-1β, and IL-6 [6,7,8] to the site, provoking intracellular phosphorylation changes of many mitogen-activated protein kinase (MAPK/ERK) mediators [9,10,11]. This acute inflammatory response to infection is predictable. However, less is known about the transition that leads to the development of chronic infections [12]. Chronic, persistent infections are challenging to treat and can present a challenge for clinicians [13]. Periprosthetic joint infection (PJI) is an infection surrounding a prosthetic knee and represents one example of localized infections that can transition into a chronic state. Dysregulation of immune mediators has been observed systemically for PJI [14,15], but the mechanisms that lead to these signaling disruptions have not been investigated [16]. PJI affects approximately 40,000 patients per year in the United States [17], and resolving these chronic infections is a high priority for clinicians. These patients suffer from chronic inflammation surrounding the joint due to presence of implant as well as infection [18,19]. This compound inflammation makes the tissue-level response difficult to understand using traditional statistical approaches. Further investigation into the tissue-level disruptions that lead to chronic infection and inflammation may allow a better understanding of how best to address these conditions.

Network analysis approaches allow for a global evaluation of these complex, tissue-level disruptions [20]. Traditional statistical methods for evaluating these contributions may be limited, as they can only evaluate one component individually. Conversely, network analysis approaches allow for an understanding of the interactions of different components with respect to the entire signaling network [20]. Currently, pathway analysis software like Ingenuity Pathway Analysis (IPA), Cytoscape, and iPathway Guide are used to analyze these types of datasets from a network perspective, and these tools offer an enriched understanding of biological networks. These applications allow users to construct networks, analyze molecular functions, and identify disease states using experimental and literature-derived data [21,22].

Beyond literature-based enrichment of data, mathematical modeling, such as network centrality parameter analysis, can be used to dissect large datasets and understand relationships between the individual components. Network centrality parameters assign quantitative values to every measured target (node) to describe how central each target is relative to all other nodes in the network. Some examples of centrality parameters are degree (number of direct neighbors), diameter (maximum distance between nodes in the network), and radiality (shortest path between a node and all other nodes, normalized to network diameter) [23].

A node with a high radiality indicates that node is central to the network, and networks with mostly high radiality nodes are behaving in an organized manner. Conversely, nodes with low centrality values have peripheral roles, and networks with many low radiality nodes may be interpreted as an open cluster of proteins that are connecting to other regulatory molecules [23]. By focusing on the nodes with low centrality outcomes, it may be possible to understand which peripheral nodes are contributing to the dysregulation observed in networks of chronic inflammation and infection that occur in TKR patients, especially those suffering from PJI. Radiality has been used in literature to probe biological networks and garner information about protein-protein interactions to understand chronic inflammatory conditions like diabetes [24], cancer [25], and chronic viral infections [26]. Ultimately, using radiality to evaluate these signaling networks allowed an opportunity to identify new therapeutic targets to combat these conditions. Evaluating the nodes that are most central and most peripheral in chronic infections like PJI may yield similar benefits.

In this study, nine cytokines and twenty-one phosphoproteins were measured in tissues surrounding the knee joint to evaluate differences between native response in primary TKA, chronic inflammatory response in aseptic TKR, and chronic infection response in septic TKR. Two tissue depths were evaluated for each group: adjacent tissue layer (ATL), unhealthy tissue that is close to the joint and requires removal; and radial tissue layer (RTL), healthy tissue that does not need to be removed. The dataset was examined using IPA and network centrality radiality to allow both qualitative and quantitative evaluations of cytokine and phosphoprotein contributions. A comparison of radiality values between primary TKA, aseptic TKR, and septic TKR allowed for a narrowing of the nodes with particularly distinct responses. These nodes may have important contributions to the disruption of normal cell signaling events. In the future, a focused analysis of these protein targets may facilitate the development of new therapeutics to combat persistent inflammation and infection observed in these patients.

## 2. Materials and Methods

### 2.1. Patients

All subjects gave informed consent for inclusion in the study, and the study was conducted in accordance with the Declaration of Helsinki. Following Institutional Review Board (IRB) approval (IRB protocol #1709745853) and patient consent, six patients undergoing primary total knee arthroplasty (TKA) and eleven patients undergoing total knee revision (TKR) procedures participated in the study (8 males and 9 females; aged 45–82 years; body max index [BMI] 24.6–43.7; information can be found in Table 1). Subjects were recruited over a 12-month period. All six primary TKA patients were undergoing elective surgery for total replacement of the knee joint with a diagnosis of osteoarthritis. In the TKR group, patients were further characterized into aseptic and septic revision procedures. Patients with aseptic revisions (N = 5) were undergoing revisions due to failures of the prosthetic joint but did not show presence of infection. Patients with septic revisions (N = 6) met clinical criteria for a PJI diagnosis, as defined by the Musculoskeletal Infection Society (MSIS) criteria [27]. All six patients diagnosed with PJI had positive tissue cultures on the day of surgery: four tested culture positive for *Staphylococcus epidermidis*, one for Methicillin-sensitive *Staphylococcus aureus* (MSSA), and one for *Enterobacter cloacae*. All groups of patients received the same pre-operative pain relief and anesthesia, per standard clinical procedures.

### 2.2. Collection of Tissue Samples

**Figure 1:**
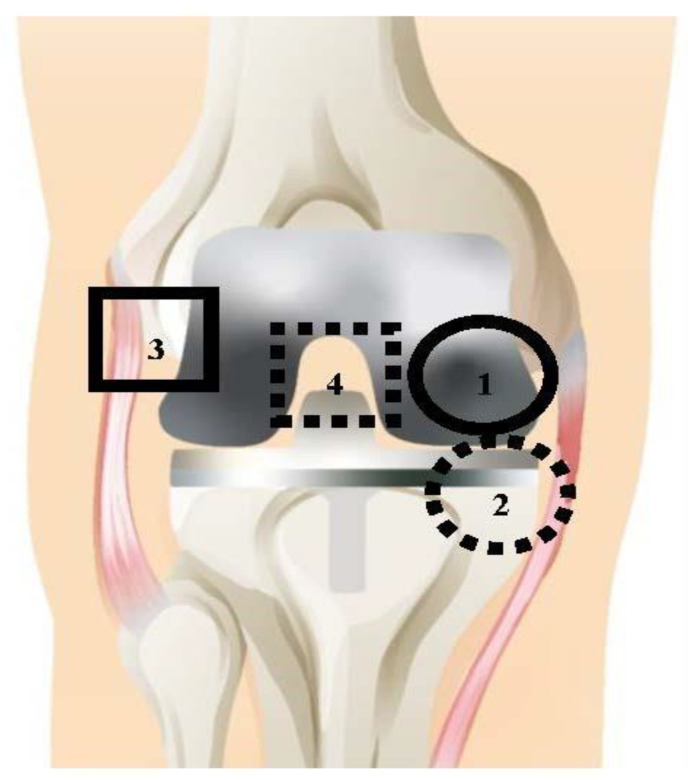
Map of approximate tissue collection locations, shown with prosthetic implant. Seven tissue samples were taken for each patient; (1) the solid circle represents the medial femoral condyle (denoted as F); (2) the dashed circle represents the medial tibial plateau (denoted as T); (3) the solid square represents the lateral gutter (denoted as LG); (4) the dashed square represents the posterior capsule (denoted as PC). Locations 1–4 were taken for the ATL layer, and locations 1–3 were taken for the RTL layer; separation between ATL (unhealthy tissue, closer to joint) and RTL (healthy tissue, further from joint) was approximately 1 cm, depending on individual patient.

All TKA and TKR procedures were performed by a single surgeon with standard debridement and washing protocols. Debridement during TKA and TKR is the removal of unhealthy tissue surrounding the joint [28]. Tissues were collected at a total of four distinct anatomical locations, shown in Figure 1. The solid line circle represents location 1: medial femoral condyle (F); the dashed line circle represents location 2: medial tibial plateau (T); the solid line square represents location 3: lateral gutter (LG); and the dashed line square represents location 4: posterior capsule (PC). These tissues were collected at two tissue layers, the adjacent tissue layer (ATL) and radial tissue layer (RTL). The ATL samples came from the initial debridement; these tissues are removed during surgery to promote better wound healing. RTL samples were taken from a tissue layer further removed from the joint after the surgeon completed debridement. The difference in depth of the RTL tissues and ATL tissues was ~1 cm. Anatomical locations 1–4 were collected for the ATL layer, and locations 1–3 were collected for the RTL layer. Location 4 (PC) could not be taken in the RTL layer due to proximity to neurovascular structures. Therefore, a total of seven tissue samples were taken for each patient.

### 2.3. Sample Preparation

Tissues were collected during TKA and TKR procedures in the operating room and immediately stored on dry ice. Once all tissues had been collected for an individual patient, they were washed with 1X cold phosphate-buffered saline (PBS) to remove blood and debris. Tissues were grossly dissected using a scalpel to remove scar tissue, then stored at −80 °C. When samples had been collected for all patients, tissues were thawed on ice and cut into sections approximately 30 mg in size; tissues were homogenized by sonication in 500 µL cell lysis solution (Bio-Rad, Hercules, CA, USA) containing 20 mM phenylmethylsulfonyl fluoride (Sigma-Aldrich, St. Louis, MO, USA). Protein extraction was performed using methods adapted from Hulse et al. [29]. Thawed samples were vortexed for 1–3 s and centrifuged at 5000× *g* for 5 min at 4 °C. The supernatant was collected and tested for total protein content using a Pierce BCA Protein Assay Kit (Thermo Scientific, Waltham, MA, USA), according to the manufacturer’s instructions. Absorbance values for total protein content were determined on an Infinite M1000 multimode plate reader (Tecan, Raleigh, NC, USA).

### 2.4. Cytokine and Phosphoprotein Measurement

To standardize samples for total protein content, tissue homogenates were individually diluted to a total protein concentration of 900 µg/mL with cell lysis buffer (Bio-Rad). Cytokine and phosphoprotein measurements were performed using magnetic bead-based multiplex Inflammation Human ProcartaPlex panel assays (Invitrogen, Carlsbad, CA, USA) and custom Bio-Plex human phosphoprotein multiplex kits. Targets were measured using a Bio-Plex 200 suspension array system and Pro II Wash Station (Bio-Rad), according to the manufacturer’s instructions (Table 2).

### 2.5. Data Processing and Statistical Analysis

Data were analyzed using Prism 5 (GraphPad, San Diego, CA, USA) and SAS JMP (Cary, NC, USA). Cytokine standard curves were generated using either a four- (4PL) or five-parameter logistic (5PL) regression model, depending on the individual protein. Cytokine concentrations are expressed as picograms of cytokine per milliliter of tissue homogenate (pg/mL). For purposes of network analyses, these values were normalized to the highest value for each cytokine. For phosphoproteins, relative phosphoprotein levels were measured via multiplex enzyme-linked immunosorbent assay (ELISA), and compared to negative control. These values were normalized to the highest value for each phosphoprotein. Contributions of cytokines and phosphoproteins were analyzed for the ATL and RTL layers. All four tissues from the ATL layer were averaged together to represent ATL depth. The three tissues from the RTL layer were averaged together to represent RTL depth. Samples with fluorescence intensity values below the lower limit of quantitation (LLOQ) or above the upper limit of quantitation (ULOQ) were omitted from statistical comparisons of cytokines and phosphoproteins. Outliers were identified using the 1.5 × interquartile range (IQR) rule and omitted from analysis [45]; these were removed on a case-by-case basis to exclude errant values that may have resulted due to assay variability. Two-way analysis of variance (ANOVA) with Bonferroni’s post-test was used to determine significant differences between primary TKA, aseptic TKR, and septic TKR tissue samples at each tissue depth, ATL and RTL. Data are expressed as the mean ± standard error of the mean (SEM). To examine any potential confounding factors in this cohort, Pearson correlations were analyzed between age, sex, and BMI and all 30 measured targets. A Bonferroni’s correction was applied, as described in [46], and the correlations were analyzed for statistical significance at *p* < 0.05. Although there are established correlations in literature between inflammatory mediators and age, sex, and BMI, there were no statistically significant correlations observed for this study, which indicates that these parameters were not confounding factors (data not shown).

### 2.6. Network Evaluation with Ingenuity Pathway Analysis (IPA)

The normalized responses were investigated with QIAGEN’s Ingenuity^®^ Pathway Analysis (IPA^®^, QIAGEN, Redwood City, CA, USA). Proposed signaling networks of cytokines and phosphoproteins were created for all groups (primary TKA, aseptic TKR, septic TKR) at the ATL depth. All networks consist of nodes from the experimental dataset and literature-derived projected nodes likely to be involved, identified by Ingenuity Knowledge Base. Up- and down-regulated responses are color coded using red and green, respectively. Briefly, IPA constructs networks building on “Focus Genes” or nodes that are highly interconnected [47]. Values from the experimental dataset influence which nodes are designated as “Focus Genes” and may alter the structure of the networks. IPA also reported top molecular and cellular functions related to the network, with corresponding scores (negative log_10_ [*p*-value of Fisher’s exact test]). The Fisher’s exact test (*p*value-) gives the likelihood of finding the identified Focus Genes by random chance in the Global Molecular Network used by IPA.

### 2.7. Network Centrality Parameter Analysis

Euclidean distances between pairs of normalized observations (cytokines and phosphoproteins) were determined for each group (primary, aseptic, septic) and depth (ATL and RTL). The definition of Euclidean distance is:

(1) $$\;\;\;E\left(\upsilon ,\omega \right)=\sqrt{\overset{n}{\underset{i=1}{\sum }}{\left(\left(normalized\;response_{\upsilon_{i}}\right)-\left(normalized\;response_{\omega_{i}}\right)\right)}^{2}\;}\;$$

where *υ* and *ω* represent the 2 responses for which the distance between is being calculated, and n signifies the replicate number. To construct networks of the relative responses of each group, Euclidean distances for each pair of nodes were used to calculate the node centrality parameter, radiality. Radiality is defined as:

(2) $$C_{rad\left(\upsilon \right)}=\frac{\sum_{\omega \epsilon N}\left(\Delta G+1-dist\left(\upsilon ,\omega \right)\right)}{n-1}$$

where ΔG represents the network (N) diameter (maximal path length of the network), dist(υ,ω) is the shortest path between a pair of nodes υ and ω, and n is the number of nodes in the network. To allow for comparisons between networks, radiality values were normalized to the average radiality for all nodes in the network. Significant radiality values were identified using a threshold value of the average radiality ± the standard deviation.

## 3. Results

### 3.1. Relative Spatial Cytokine Responses

**Figure 2:**
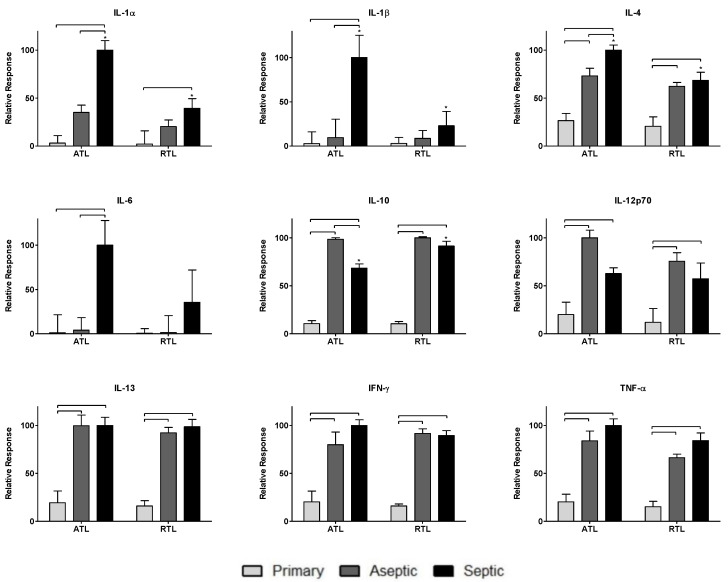
Relative cytokine levels measured in tissues from primary TKA, aseptic TKR, and septic TKR at adjacent tissue layer (ATL) and radial tissue layer (RTL) debridement depths. Relative cytokine responses (normalized to highest cytokine signal) were observed for all three patient groups: primary, aseptic, and septic at two debridement depths: ATL is closer to the knee joint, and RTL is approximately 1 cm removed from the knee joint. Statistically significant differences (*p* < 0.05) were determined by two-way ANOVA with Bonferroni’s post-test to examine group-dependent and spatially-dependent differences in cytokine relative response. Differences for the same group (i.e., septic) between ATL and RTL are marked with an asterisk (*). Differences between groups within a tissue layer are denoted with bars. Responses are shown as the mean ± SEM.

Nine cytokines were measured in this study: IL-1α, IL-1β, IL-4, IL-6, IL-10, IL-12p70, IL-13, IFN-γ, and TNF-α. The responses of these cytokines are shown in Figure 2. Cytokine levels were normalized across groups (primary TKA, aseptic TKR, septic TKR) and debridement depths (ATL, RTL) to the highest value for each cytokine. Normalizing by this method is important to appropriately weight cytokines equally for network analysis rather than relying on raw concentrations. This weighting is performed to understand the contributions of each node to the network, relative to other nodes. Group-dependent differences were observed, as were spatial differences between debridement depths. Briefly, the aseptic TKR and septic TKR groups had higher cytokine responses than the primary TKA group for all cytokines. IL-1α, IL-1β, IL-4, and IL-6 had higher levels in septic TKR than aseptic TKR at a statistically significant level (*p* < 0.05). IL-10 was the only cytokine with a lower relative response in the septic TKR when compared to aseptic TKR at a statistically significant level (*p* < 0.05). IL-12p70 seemed to show the same trend, but was not significant at *p* < 0.05. There were also differences between ATL and RTL in septic TKR tissues. For IL-1α, IL-1β, and IL-4, there were statistically significant differences between ATL and RTL depths for the septic group (*p* < 0.05).

### 3.2. Relative Spatial Phosphoprotein Responses

**Figure 3:**
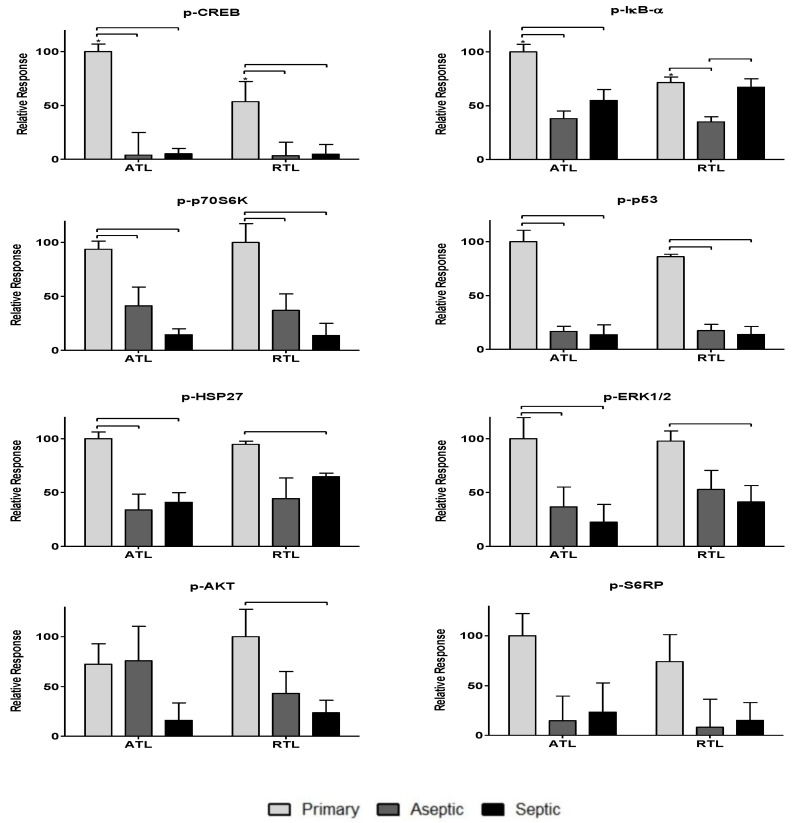
Relative levels of phosphoproteins associated with the proliferative processes in acute wound healing. Relative phosphoprotein responses (normalized to highest signal) were observed for all three patient groups: primary, aseptic, and septic at two debridement depths: ATL is closer to the knee joint, and RTL is approximately 1 cm removed from the knee joint. Statistically significant differences (*p* < 0.05) were determined by two-way ANOVA with Bonferroni’s post-test to examine group-dependent and spatially-dependent differences in protein phosphorylation. Differences for the same group (i.e., septic) between ATL and RTL are marked with an asterisk (*). Differences between groups within a tissue layer are denoted with bars. Responses are shown as the mean ± SEM.

**Figure 4:**
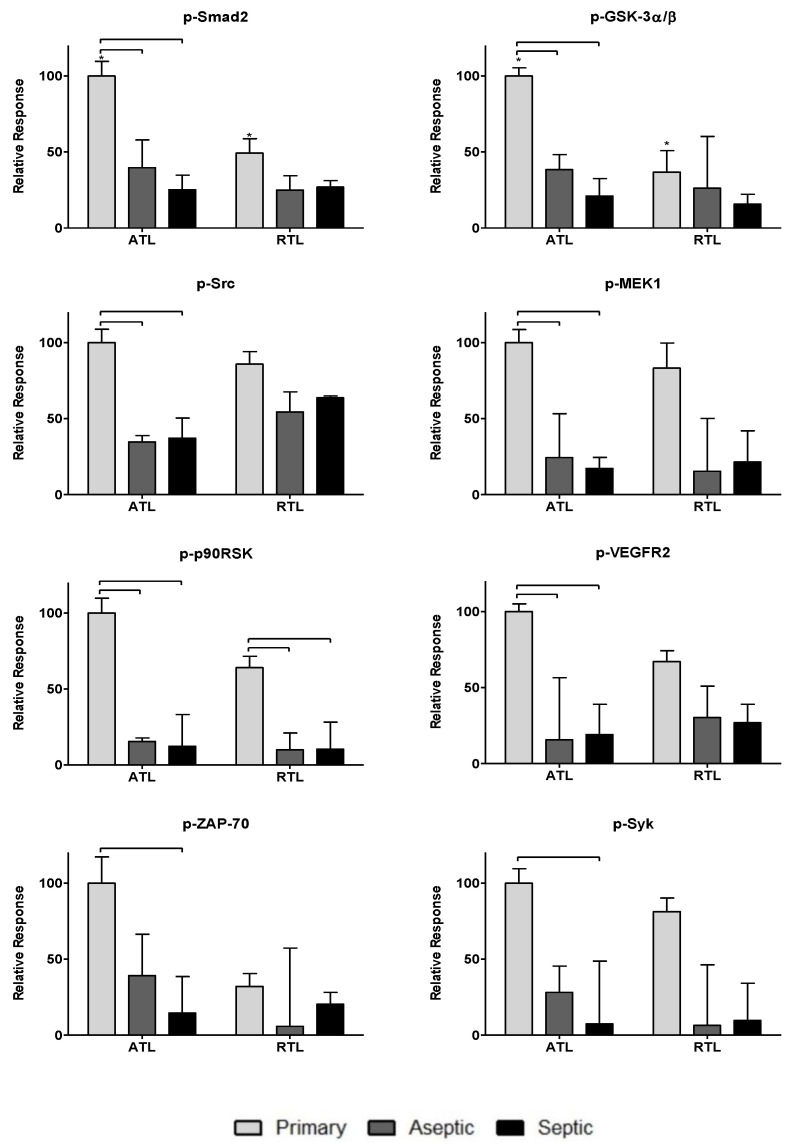
Relative phosphoprotein levels associated with cell migration processes in acute wound healing. Relative phosphoprotein responses (normalized to highest signal) were observed for all three patient groups: primary, aseptic, and septic at two debridement depths: ATL is closer to the knee joint, and RTL is approximately 1 cm removed from the knee joint. Statistically significant differences (*p* < 0.05) were determined by two-way ANOVA with Bonferroni’s post-test to examine group-dependent and spatially-dependent differences in protein phosphorylation. Differences for the same group (i.e., septic) between ATL and RTL are marked with an asterisk (*). Differences between groups within a tissue layer are denoted with bars. Responses are shown as the mean ± SEM.

**Figure 5:**
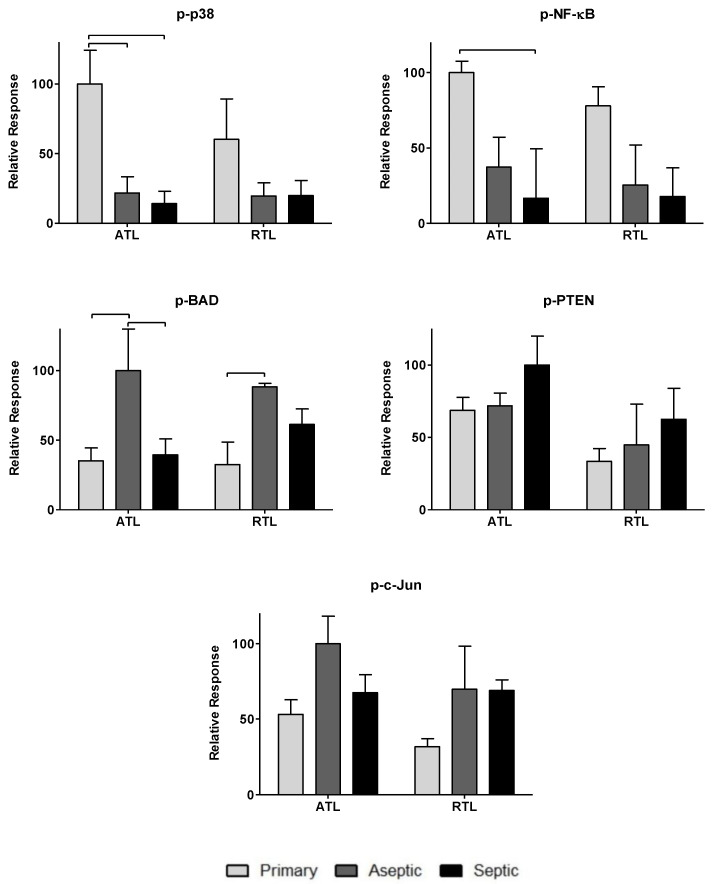
Relative levels of pro-apoptotic and inhibitory wound healing phosphoproteins in acute wound healing. Relative phosphoprotein responses (normalized to highest signal) were observed for all three patient groups: primary, aseptic, and septic at two debridement depths: ATL is closer to the knee joint, and RTL is approximately 1 cm removed from the knee joint. Statistically significant differences (*p* < 0.05) were determined by two-way ANOVA with Bonferroni’s post-test to examine group-dependent and spatially-dependent differences in protein phosphorylation. Differences between groups within a tissue layer are denoted with bars. Responses are shown as the mean ± SEM.

To further investigate the impact of the observed cytokines on tissue response, twenty-one phosphoproteins were measured: p-CREB, p-HSP27, p-IκB-α, p-MEK1, p-S6RP, p-Smad2, p-Src, p-Syk, p-c-Jun, p-AKT, p-p53, p-p38, p-p70S6K, p-PTEN, p-ZAP-70, p-BAD, p-ERK1/2, p-GSK-3α/β, p-p90RSK, pVEGFR2, and p-NF-κB (more information can be found in Table 2). The data are spread over Figure 3, Figure 4 and Figure 5. Figure 3 includes phosphoproteins most associated with proliferative wound healing processes [30,36,37,39]. Phosphoproteins in Figure 4 have roles in cell migration and fibrotic processes [2,30,32,34,35,40,41]. Finally, Figure 5 includes the phosphoproteins that have pro-apoptotic roles and have been associated with delayed wound healing through their involvement in muscle catabolism [33,38,39,40]. Most phosphoproteins exhibited higher responses in the primary TKA tissues than in aseptic TKR and septic TKR tissues, for both ATL and RTL depths, and many exhibited group-dependent differences, especially in ATL depth. Some exceptions to this trend were p-c-Jun and p-BAD, which had the highest responses in aseptic TKR, then septic TKR, followed by primary TKA; also, p-PTEN showed the highest response in septic tissues (Figure 5). Specific group-dependent comparisons are shown in Figure 3, Figure 4 and Figure 5.

Tissue depths were also compared for phosphoproteins. Responses in the ATL were higher than responses in the RTL for most phosphoproteins. However, several proteins showed notably higher levels in RTL than ATL for at least one of the three tissue groups: p-BAD, p-Src, p-IκB-α, p-HSP27, p-ERK1/2, and p-VEGFR2 (Figure 3, Figure 4 and Figure 5). Comparisons of ATL vs. RTL for each group are shown in Figure 3, Figure 4 and Figure 5.

### 3.3. IPA-Generated Networks

**Figure 6:**
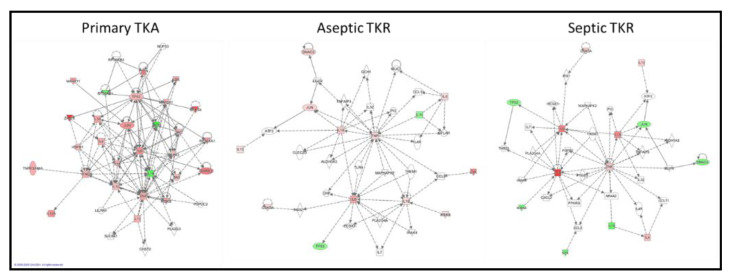
Ingenuity Pathway Analysis (IPA)-generated networks for primary TKA, aseptic TKR, and septic TKR groups based on cytokine and phosphoprotein datasets. Proposed networks used relative cytokine and phosphoprotein responses in the ATL depth, illustrating the differences in tissue responses for the three groups. The nodes are illustrated in a “heat map” coloring scheme, with red denoting up-regulation, green denoting down-regulation, and the intensity of color correlates to the intensity of relative response. The networks are supplemented with other nodes likely to be involved, as identified in the Ingenuity Knowledge Base. A solid line represents a direct interaction between two nodes, while a dotted line denotes an indirect relationship.

Networks for the three groups (primary TKA, aseptic TKR, and septic TKR) were constructed from the same set of cytokines and phosphoproteins for the ATL layer. The network connectivity varied greatly between the three groups (Figure 6). Qualitatively, the primary TKA network showed higher connectivity and more experimentally validated up- and down-regulation of targets, as shown by the red and green coloring, respectively. Further, the connections between targets, also known as “edges,” varied between the three groups. Edges denote connections between nodes; in IPA, direct relationships are shown by solid lines, and indirect relationships are shown by dotted lines. The primary TKA network showed 139 edges; 23 of these edges were direct, and 116 were indirect. For aseptic TKR, 65 total edges were identified: 4 direct, 61 indirect. For septic TKR, 61 total edges are shown: 4 direct and 57 indirect. IPA uses the experimental dataset to identify related IPA networks, shown in Table 3. A p-score is shown for each IPA network match, and the p-score is calculated based on the -log_10_(*p*-value) for the Fisher’s exact test. A higher IPA p-score indicates a stronger match; p scores above 21 are generally considered good matches [48].

### 3.4. Normalized Radiality of All 30 Nodes

**Figure 7:**
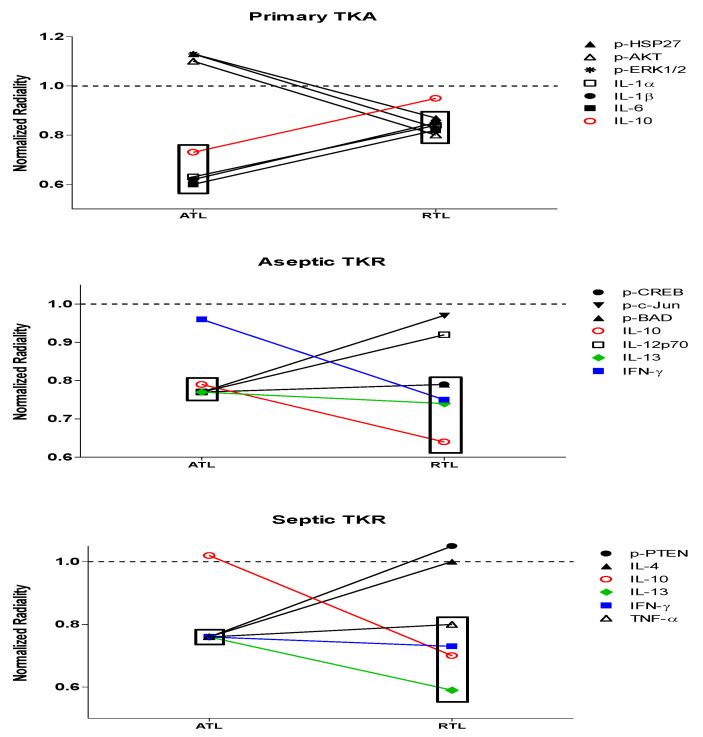
Changes in significant nodes between groups for low radiality outcomes. Nodes with low radiality outcomes that differed between primary TKA response and aseptic/septic TKR responses are shown (significance threshold: the average radiality ± standard deviation). Boxes indicate significance at varying depths. IL-10 is shown in red to highlight its presence in all three groups: primary TKA, aseptic TKR, and septic TKR. IL-13 (green) and IFN-γ (blue) are also colored to highlight overlap in both aseptic TKR and septic TKR groups.

Based on the ANOVA data and IPA-generated networks, all of these cytokine and phosphoprotein targets have roles to play in both infection response and wound healing. To further understand the most important targets, network centrality parameter analysis was performed by analyzing a network centrality parameter, radiality. Radiality values were determined for each cytokine and phosphoprotein node and normalized to the average radiality for the network (e.g., primary TKA, ATL layer). These values are presented in Table 4 and Table 5. Changes in significant radiality outcomes can allow for a better understanding of the “drivers” of each network and deviations from normal response (Figure 7). Nodes with significant radiality values are bolded; the significance threshold used was the average radiality ± standard deviation. Based on previous work [49], we expect significant radiality outcomes with low radiality values to be the most likely drivers of the dysregulation for persistent inflammation and infection of aseptic and septic TKR, respectively.

While several nodes were significant within each of the six networks, respectively, some nodes showed a group-dependent trend in significance (Table 4 and Table 5). There were changes in significance between the native primary TKA response and aseptic or septic TKR responses. In the primary TKA networks, IL-1α, IL-1β, IL-6, and IL-10 gave significant low radiality outcomes for the ATL; p-HSP27, p-AKT, p-ERK1/2, IL-1α, IL-1β, and IL-6 were significant in the RTL. Differences for the aseptic TKR group include p-CREB, p-c-Jun, p-BAD, IL12p70, and IL-13 in the ATL; p-CREB, p-BAD, IL-10, IL-13, and IFN-γ for the RTL. Deviations in the septic TKR group include p-PTEN, IL-4, IL-13, IFN-γ, and TNF-α in the ATL layer and IL-10, IL-13, IFN-γ, and TNF-α in the RTL layer.

## 4. Discussion

The cytokine and phosphoprotein targets measured in this study are known to be significant contributors to inflammatory responses in general [2], but the interconnected relationships of these targets remain to be elucidated for PJI. Further, many of these targets have not been studied on a tissue level for chronic inflammation and infection, so much of the dysregulation that occurs in immune response and wound healing processes remains unknown [12]. Relative cytokine and phosphoprotein responses were measured to understand the trends in response across three groups of patients: primary TKA, aseptic TKR, and septic TKR at two tissue depths: ATL and RTL.

Higher relative cytokine levels were observed in either aseptic or septic TKR samples compared to primary TKA tissues. IL-1α, IL-1β, IL-4, and IL-6 showed infection-specific relative responses, with higher levels in septic TKR than both aseptic TKR and primary TKA (*p* < 0.05, Figure 2). These cytokines have been identified in literature as important early immune response mediators in PJI [50]. Additionally, there were spatial differences between ATL and RTL layers for IL-1α, IL-1β, IL-4, and IL-10 (Figure 2). The spatial discrepancies observed in this study suggested that the cytokine response is more robust in the ATL layer of septic tissues compared to the RTL. The spatial relationships were unclear for primary TKA and aseptic TKR using ANOVA comparisons (Figure 2).

Phosphoproteins were also included in this analysis as many hold central roles in early infection response [4]. The phosphoproteome has not been thoroughly investigated for chronic joint inflammation and infection in PJI, but the relationships between cytokines and phosphoproteins may reveal important information considering the central role of these signaling proteins in cell cycle regulation [9], cell proliferation [36], inflammatory processes [49], and wound healing [30]. Most phosphoproteins were found in higher levels in primary TKA tissues (Figure 3, Figure 4 and Figure 5). While the septic TKR gave the highest response of most cytokines, it often showed the lowest levels of phosphoproteins (Figure 3, Figure 4 and Figure 5). While many of the phosphoproteins tested are downstream targets of cytokines [2,32,33,38,40,41,42,43], decreased levels of wound healing-associated phosphoproteins have previously been observed in other studies [14,15]. Notable exceptions were p-c-Jun and p-BAD, which were highest in aseptic TKR, and p-PTEN, which was highest in septic TKR (Figure 5). All three of these phosphoproteins have associated pro-apoptotic functions in acute wound healing [33,38,39], which may be related to their increased phosphorylation in aseptic and septic TKR tissues, respectively. Phosphoprotein levels also showed spatial trends between ATL and RTL at a statistically significant level (*p* < 0.05) for p-IκB-α, p-GSK-3α/β, p-Smad2, and p-CREB (Figure 3 and Figure 4). All four of these phosphoproteins are related to cell migration and proliferation, and have important roles for wound healing [30,32,34]. The results of this study showed higher levels for these phosphoproteins in the ATL of primary TKA, compared to RTL of primary TKA, which suggests tissues closer to the joint have increased wound healing activity (Figure 3 and Figure 4).

While traditional ANOVA comparisons gave information about the relative responses of cytokines and phosphoproteins, chronic inflammation and infection involve a series of deeply interconnected targets [3,12], which makes it difficult to fully understand the tissue responses when only considering each target in isolation. The ANOVA data alone do not fully explain which targets may be contributing most to the disruptions in responses observed in aseptic and septic TKR. IPA analysis was used to comparatively assess the connectivity between the three groups. IPA has proven to be a useful tool for visualizing the connectivity of different nodes (i.e., genes, proteins, etc.) involved in networks [51]. Figure 6 illustrates the utility of IPA for comparing different networks qualitatively and depicts the differences between primary TKA, aseptic TKR, and septic TKR networks for each of the ATL layers. The primary TKA shows better connectivity between targets than aseptic TKR and septic TKR, suggesting there may be dysregulation occurring in both aseptic and septic TKR tissues (Figure 6). Additionally, Table 3 lists the top IPA network hits for each of the three networks. For proteomic analysis, a p-score above 21 is considered a good match [48]. Only the primary TKA network was able to make a match above this threshold. Based on the IPA analysis, both aseptic TKR and septic TKR networks show a lack of connectivity compared to primary TKA, which may prevent a reliable IPA network match (Table 3).

A network centrality approach was also utilized to quantitatively assess which targets were close to (high radiality) or distant from (low radiality) the center of each of the networks. Radiality comparisons may reveal the most likely nodes contributing to the dysregulation observed in the IPA networks. Based on previous work in a rodent model of trauma [49], we expect that differences in nodes with low radiality between primary TKA response and aseptic or septic TKR responses may indicate the most likely causes of disruptions to normal signaling. In this study, low radiality outcomes were the most likely contributors to cell signaling dysregulation leading to chronic inflammation and infection. A significance threshold of the average radiality ± standard deviation was used to denote significant cytokine and phosphoprotein nodes (Table 4 and Table 5). Differences existed in significant nodes across groups and between depths.

The primary TKA group represents the native response, as these tissues are not in contact with implants or infection that cause persistent inflammation [52,53]. In primary TKA, all significant nodes in the ATL had low radiality values, and all four were cytokines: IL-1α, IL-1β, IL-6, and IL-10. Within this network, these cytokines appear to be acting as regulatory molecules. IL-1α, IL-1β, and IL-6 are all pro-inflammatory cytokines vital for early inflammatory immune response [6,8]. The anti-inflammatory IL-10 is central for wound resolution [54]. In the RTL of primary TKA, nodes with significant low radiality values were p-HSP27, p-AKT, p-ERK1/2, IL-1α, IL-1β, and IL-6 (Table 4 and Table 5). This suggests that there is still a significant contribution of pro-inflammatory cytokines in healthy tissues spatially removed from the joint. p-HSP27, p-AKT, and p-ERK1/2 have all been linked to early proliferative wound healing responses in trauma [55] and skin wounds [56]. Their low radiality outcomes suggested that these three phosphoproteins may be driving the tissue healing response. Additionally, in the RTL of primary TKA, seven phosphoproteins had significantly high radiality values (Table 4), suggesting that there is an organized wound healing response in tissues further away from the joint.

The aseptic and septic TKR groups were compared to the primary TKA group to understand differences in radiality outcomes. In the aseptic ATL, nodes with significant low radiality outcomes were p-CREB, p-c-Jun, p-BAD, IL-6, IL-10, IL-12p70, and IL-13. Additionally, eight phosphoproteins and one cytokine had significant high radiality outcomes (Table 3). Overall, in the ATL of aseptic TKR, there appears to be a balance of regulated and dysregulated healing processes. In combination with the IPA network results, this suggested that dysregulation may be caused by reduced contributions for pro-inflammatory IL-1α and IL-1β and an increased role for anti-inflammatory IL-13 between primary TKA response and aseptic TKR response at the joint (Figure 6). The pro-apoptotic actions of peripheral p-c-Jun and p-BAD [33,39], and inactivation of CREB [57] in aseptic TKR could also be driving these disruptions (Figure 6 and Figure 7). In the RTL of aseptic TKR, p-CREB, p-BAD, IL-10, IL-13, and IFN-γ gave significant low radiality outcomes (Table 3 and Table 4). The aseptic RTL tissues showed a shift to all significant nodes showing low radiality outcomes (Table 4). Compared to the primary RTL, there is a notable induction of anti-inflammatory cytokines IL-10, IL-13, and IFN-γ. Significance of p-CREB and p-BAD suggested these activated proteins may be promoting apoptosis [30,39] in presumably healthy aseptic tissues. Further, the coordinated healing response observed in primary RTL tissues is no longer present, as there were no significant high radiality outcomes in aseptic RTL (Table 4). Even in the clinically “healthy” tissues for the aseptic group, there is a large amount of dysregulation present, and it appears to be primarily driven by these seven targets: p-c-Jun, p-CREB, p-BAD, IL-10, IL-12p70, IL-13, and IFN-γ.

In septic TKR, nodes with significant low radiality outcomes in the ATL were p-PTEN, IL-1α, IL-1β, IL-4, IL-6, IL-13, IFN-γ, and TNF-α. Notably, there were no significant high radiality outcomes (Table 3). While this may somewhat reflect the strong cytokine-dependent response observed in primary TKA, differences include increased contributions of anti-inflammatory IL-4, IL-13, and IFN-γ, pro-inflammatory TNF-α, and pro-apoptotic p-PTEN in the septic TKR group. The ATL of septic TKR showed a notable induction of anti-inflammatory cytokines not observed in the primary TKA. In the septic RTL layer, IL-10, IL-13, IFN-γ, and TNF-α gave significant low radiality values. There were no significant outcomes with high radiality values in this network (Table 4). Additionally, there was no overlap in significant low radiality targets between primary TKA and septic TKR tissues at the RTL depth. This loss of centrality for wound healing targets in the “healthy” septic TKR tissues reflects a disruption in normal response.

There were some limitations to the study. A single surgeon collected all tissue samples for the cohort of patients involved. Treatment of PJI via debridement is a subjective assessment of tissue viability [28], so the delineation between “healthy” and “unhealthy” tissues may vary between surgeons. The results for the RTL depths of aseptic and septic TKR highlighted the disruptions still present in presumably healthy tissues removed from the joint, so a larger cohort of patients from different surgeons may aid future studies in analyzing these targets. Further, it is difficult to fully disentangle the inflammation present in native response from chronic inflammation and infection. The primary TKA group is expected to experience inflammation as a result of the surgery [58], which is why this study focused on outlining the differences between groups. These differences may not account for all inflammation occurring in the tissues, but the discrepancies between targets may help identify the dysregulation observed in aseptic and septic TKR. Differences in tissue composition (including bone, cartilage, and synovium) may also have played a role in introducing variability between cytokine and phosphoprotein levels; this study focused on including the most likely tissues taken from debridement, regardless of composition. Finally, the IPA analysis was only qualitatively useful in this case due to experimental constraints. While IPA can be used quantitatively for proteomics [59], the samples must be normalized to a control group. The primary TKA is not a true control, only a comparative group. In human subjects, we cannot ethically collect a true tissue control (i.e., healthy individuals with no inflammation present), which limited our ability to analyze via IPA. However, the qualitative comparison at the joint still supported the network centrality analysis, and the IPA provided some confirmation of the roles of the targets involved.

## 5. Conclusions

The acute intra- and extracellular responses to infection have been studied extensively, and these studies have provided valuable information for clinicians to develop diagnostics and therapeutics to combat these infections [50]. However, less is known about the dysregulation that occurs when inflammation and infections become chronic, which is the case in localized infections like PJI [12]. In this study, we aimed to define the impact of individual cytokines and phosphoproteins on chronic inflammation and infection in PJI using a network centrality parameter approach. Overall, network centrality analysis showed the native response in primary TKA tissues was dictated by a balance of pro- and anti-inflammatory cytokines. Tissues in the ATL were highly influenced by pro-inflammatory cytokines IL-1α, IL-1β, and IL-6 and anti-inflammatory IL-10. A variety of pro-inflammatory cytokines and wound healing phosphoproteins were central to the network in the RTL, and this response was reflective of normal tissue healing processes [8,30,43] (Table 3). Deviations from this response were observed in both aseptic and septic TKR groups. In aseptic TKR tissues, a shift to increased peripheral roles for pro-apoptotic and anti-inflammatory targets was prevalent at both ATL and RTL tissue depths. In the septic ATL layer, pro-apoptotic p-PTEN and anti-inflammatory cytokines IL-4, IL-13, and IFN-γ showed significant losses of centrality compared to primary TKA. The high contributions of nodes with seemingly contradictory roles, combined with the loss of overall IPA network connectivity, highlights the dysregulation near the joint in septic TKR tissues. At the septic RTL depth, anti-inflammatory cytokines dominated the response, showing a hallmark absence of coordinated phosphoproteins linked to wound healing. The radiality data as a whole suggested that disrupted signaling pathways are present for both aseptic and septic TKR, even in presumably “healthy” tissues. Targeting the proteins with significant radiality outcomes in chronic inflammation and infection may prove useful for developing more effective therapeutics, and future studies should focus on these proteins to promote tissue healing and infection resolution in PJI.

## Figures and Tables

**Table 1:** Patient Information. Six primary total knee arthroplasty (TKA) and eleven revision total knee revision (TKR) patients were enrolled in the study, creating a heterogenous cohort of males and females varying in age (45–82 years) and comorbidities. Primary TKA patients have ID format P#; revision TKR patients have ID format F#. This table lists general patient information including the pathogen for which each septic patient tested positive on the day of surgery. Serum C-reactive protein (CRP) values were obtained pre-operatively in the revision setting. Cultures were obtained from intraoperative tissue samples.

ID	Sex	TKA/TKR	BMI (kg/m^2^)	Diabetic (Y/N)	CRP (mg/L)	Culture
P1	F	TKA	33.8	N	N/A	Negative
P2	F	TKA	39.8	N	N/A	Negative
P3	F	TKA	39.8	N	N/A	Negative
P4	M	TKA	29.7	Y	N/A	Negative
P5	M	TKA	24.6	N	N/A	Negative
P6	M	TKA	27.2	N	N/A	Negative
F1	F	TKR—Aseptic	28.2	N	4.3	Negative
F2	F	TKR—Aseptic	29.8	N	0.2	Negative
F3	F	TKR—Aseptic	33.9	N	<1	Negative
F4	M	TKR—Aseptic	40.4	Y	3.6	Negative
F5	M	TKR—Aseptic	26.2	N	2.1	Negative
F6	F	TKR—Septic	43.7	N	28.8	*S. epidermidis*
F7	F	TKR—Septic	30.8	Y	161.4	*S. epidermidis*
F8	F	TKR—Septic	41.9	N	21.7	*E. cloaecae*
F9	M	TKR—Septic	36.2	N	33.5	*MSSA*
F10	M	TKR—Septic	33.8	Y	3.8	*S. epidermidis*
F11	M	TKR—Septic	31.9	N	111.9	*S. epidermidis*

**Table 2:** Cytokines and Phosphoprotein Targets Measured in Tissue Samples. Citations are noted in brackets.

	Target	Relevant Functions in Acute Wound Healing Response
**Phosphoprotein (site)**	p-CREB (Ser133)	Inhibition of CREB via phosphorylation promotes wound closure [30]
	p-HSP27 (Ser78)	Activation of HSP27 may inhibit stress-induced apoptosis [31]
	p-IκBα (Ser32/Ser36)	Pro-wound healing, inhibits actions of NF-κB [32]
	p-MEK1 (Ser217/Ser221)	Essential for migration of epithelial layers [33]
	p-S6RP (Ser235/Ser236)	Activated during proliferative growth phase [30]
	p-Smad2 (Ser465/Ser467)	Regulates keratinocyte migration during proliferation [34]
	p-Src (Tyr416)	Promotes keratinocyte migration in wound healing [32]
	p-Syk (Tyr352)	Important for cellular migration in wound healing [35]
	p-c-Jun (Ser63)	Induces apoptosis of immune cells in skin wound healing [33]
	p-AKT (Ser473)	Phosphorylation of AKT promotes wound closure [30]
	p-p53 (Ser15)	Activated p53 accelerates cutaneous wound healing by increasing cell proliferation [36]
	p-p38 (Thr180/Tyr182)	Activated p38 involved in muscle catabolism [32]
	p-p70S6K (Ser380)	Growth factor associated with cell proliferation [37]
	p-PTEN (Ser380)	Pro-apoptotic, inhibits acute wound healing [38]
	p-ZAP-70 (Tyr319)	Stimulates cell migration during wound healing [35]
	p-BAD (Ser136)	Phosphorylation of BAD activates pro-apoptotic functions [39]
	p-ERK1/2 (Thr202/Tyr204)	Important for early proliferative response in wound healing [37]
	p-GSK-3α/β (Ser21/Ser9)	Controls wound healing and fibrosis progression [30]
	p-p90RSK (Ser380)	Downstream effector of MEK/ERK pathway in wound healing, regulator of cell migration [40]
	p-VEGFR2 (Tyr1175)	Stimulates angiogenic cascade during re-epithelialization [41]
	p-NF-κB p65 (Ser536)	Linked to muscle atrophy and catabolism [32]
**Cytokine**	IL-1β	Early initiator of infection-driven inflammation [2]
	IL-4	Anti-inflammatory cytokine that activates Stat6, suppressing cell death [42]
	IL-6	Initiator of early inflammatory response to implants and infection [2]
	IL-1α	Early recruitment of immune cells in response to infection [2]
	IL-10	Down-regulator of several inflammatory cytokines (i.e., IL-1, IL-6, IL-12, IFN-γ, TNF-α) [43]
	IL-12p70	Pro-inflammatory cytokine involved in adaptive immunity, produced by activated immune cells [43]
	IL-13	Th2-associated cytokine critical in tissue remodeling [44]
	IFN-γ	Anti-inflammatory cytokine that has been associated with inhibition of wound healing [43]
	TNF-α	Early pro-inflammatory mediator of inflammation [2]

**Table 3:** Top 2 IPA Networks for Primary TKA, Aseptic TKR, and Septic TKR Groups. Network p-scores are calculated by IPA using the negative log_10_ (*p*-value) of Fisher’s exact test. The p-value describes the probability of finding the cytokines/phosphoproteins randomly in the databases utilized by IPA to construct the network. Networks with p-scores above the threshold of 21 are bolded.

Primary TKA		Aseptic TKR		Septic TKR	
IPA Network	p-Score	IPA Network	p-Score	IPA Network	p-Score
**Cell-mediated immune response, cellular development, cellular function and maintenance**	**72**	Inflammatory response, cellular movement, cell death and survival	16	Cellular movement, inflammatory response, hematological development and function	16
Cancer, organismal injury and abnormalities, cell cycle	2	Cell-mediated immune response, cellular development, cellular function and maintenance	9	Cell death and survival, organismal injury and abnormalities, cellular development	9

**Table 4:** Normalized Radiality of Nodes in the ATL Layer. Significant target values for each individual network are bolded (significance threshold: the average radiality ± standard deviation).

Node	ATL Primary TKA	ATL Aseptic TKR	ATL Septic TKR
p-CREB	0.96	**0.77**	1.15
p-HSP27	1.13	1.14	1.15
p-IκBα	1.13	**1.16**	1.10
p-MEK1	1.13	1.08	1.10
p-S6RP	1.13	0.98	1.13
p-Smad2	1.13	**1.15**	1.14
p-Src	1.13	**1.15**	1.15
p-Syk	1.13	1.11	0.95
p-c-Jun	1.04	**0.77**	1.03
p-AKT	1.10	0.99	1.08
p-p53	1.13	1.00	1.06
p-p38	1.13	1.05	1.06
p-p70SK6	1.13	**1.15**	1.07
p-PTEN	1.09	1.02	**0.76**
p-ZAP-70	1.13	**1.16**	1.07
p-BAD	0.96	**0.77**	1.15
p-ERK1/2	1.13	**1.15**	1.13
p-GSK-3a/b	1.13	**1.16**	1.12
p-p90RSK	1.13	0.99	1.04
p-VEGFR2	1.13	0.99	1.11
p-NF-kB	1.13	**1.16**	1.09
IL-1b	**0.62**	0.91	**0.76**
IL-4	0.90	1.01	**0.76**
IL-6	**0.60**	**0.82**	**0.76**
IL-1a	**0.63**	**1.15**	**0.76**
IL-10	**0.73**	**0.79**	1.02
IL-12p70	0.84	**0.77**	1.06
IL-13	0.84	**0.77**	**0.76**
IFN-y	0.85	0.96	**0.76**
TNF-a	0.85	0.93	**0.76**

**Table 5:** Normalized Radiality of Nodes in the RTL Layer. Significant target values for each individual network are bolded (significance threshold: the average radiality ± standard deviation).

Node	RTL Primary TKA	RTL Aseptic TKR	RTL Septic TKR
p-CREB	**1.12**	**0.79**	1.06
p-HSP27	**0.87**	1.12	1.03
p-IκBα	1.07	1.13	1.01
p-MEK1	0.99	1.05	1.08
p-S6RP	1.06	0.98	1.02
p-Smad2	**1.12**	1.11	1.10
p-Src	0.97	1.08	1.04
p-Syk	1.01	0.96	0.94
p-c-Jun	**1.11**	0.97	0.99
p-AKT	**0.80**	1.12	1.09
p-p53	0.97	1.07	1.00
p-p38	1.11	1.08	1.06
p-p70SK6	**0.80**	1.13	1.00
p-PTEN	**1.12**	1.12	1.05
p-ZAP-70	**1.11**	0.96	1.07
p-BAD	**1.12**	**0.79**	1.06
p-ERK1/2	**0.83**	1.09	1.11
p-GSK-3a/b	**1.12**	1.12	1.02
p-p90RSK	1.10	1.00	0.95
p-VEGFR2	1.09	1.13	1.10
p-NF-kB	1.04	1.12	1.04
IL-1b	**0.85**	0.99	1.08
IL-4	1.04	1.03	1.00
IL-6	**0.82**	0.89	1.11
IL-1a	**0.84**	1.09	1.11
IL-10	0.95	**0.64**	**0.70**
IL-12p70	0.96	0.92	1.07
IL-13	1.01	**0.74**	**0.59**
IFN-y	1.01	**0.75**	**0.73**
TNF-a	1.00	1.00	**0.80**

## References

[1] Arango Duque G., Descoteaux A. (2014). Macrophage cytokines: Involvement in immunity and infectious diseases. Front. Immunol..

[2] Chaudhry H., Zhou J., Zhong Y., Ali M.M., McGuire F., Nagarkatti P.S., Nagarkatti M. (2013). Role of cytokines as a double-edged sword in sepsis. In Vivo.

[3] Cavaillon J.M., Adib-Conquy M., Fitting C., Adrie C., Payen D. (2003). Cytokine cascade in sepsis. Scand. J. Infect. Dis..

[4] Richter E., Mostertz J., Hochgräfe F. (2016). Proteomic discovery of host kinase signaling in bacterial infections. Proteomics Clin. Appl..

[5] Zhang H., Sun J., Ye J., Ashraf U., Chen Z., Zhu B., He W., Xu Q., Wei Y., Chen H. (2015). Quantitative label-free phosphoproteomics reveals differentially regulated protein Phosphorylation involved in West Nile virus-induced host inflammatory response. J. Proteome Res..

[6] Ma L., Zhang H., Yin Y.L., Guo W.Z., Ma Y.Q., Wang Y.B., Shu C., Dong L.Q. (2016). Role of interleukin-6 to differentiate sepsis from non-infectious systemic inflammatory response syndrome. Cytokine.

[7] Lopez-Castejon G., Brough D. (2011). Understanding the mechanism of IL-1β secretion. Cytokine Growth Factor Rev..

[8] Dinarello C.A. (2018). Overview of the IL-1 family in innate inflammation and acquired immunity. Immunol. Rev..

[9] Seah C.C., Phillips T.J., Howard C.E., Panova I.P., Hayes C.M., Asandra A.S., Park H.Y. (2005). Chronic wound fluid suppresses proliferation of dermal fibroblasts through a Ras-mediated signaling pathway. J. Investig. Dermatol..

[10] Kaminska B. (2005). MAPK signalling pathways as molecular targets for anti-inflammatory therapy—From molecular mechanisms to therapeutic benefits. Biochim. Biophys. Acta.

[11] Rämet M., Lanot R., Zachary D., Manfruelli P. (2002). JNK signaling pathway is required for efficient wound healing in Drosophila. Dev. Biol..

[12] Brady R.A., Mocca C.P., Plaut R.D., Takeda K., Burns D.L. (2018). Comparison of the immune response during acute and chronic Staphylococcus aureus infection. PLoS ONE.

[13] Li C., Renz N., Trampuz A. (2018). Management of periprosthetic joint infection. Hip Pelvis.

[14] Seebach E., Kubatzky K.F. (2019). Chronic implant-related bone infections—Can immune modulation be a therapeutic strategy?. Front. Immunol..

[15] Lüthje F.L., Jensen L.K., Jensen H.E., Skovgaard K. (2020). The inflammatory response to bone infection—A review based on animal models and human patients. APMIS.

[16] Grant S.S., Hung D.T. (2013). Persistent bacterial infections, antibiotic tolerance, and the oxidative stress response. Virulence.

[17] Tande A.J., Patel R. (2014). Prosthetic joint infection. Clin. Microbiol. Rev..

[18] Kalore N.V., Gioe T.J., Singh J.A. (2011). Diagnosis and management of infected total knee arthroplasty. Open Orthop. J..

[19] Dyskova T., Kriegova E., Slobodova Z., Zehnalova S., Kudelka M., Schneiderova P., Fillerova R., Gallo J. (2019). Inflammation time-axis in aseptic loosening of total knee arthroplasty: A preliminary study. PLoS ONE.

[20] Morel P.A., Lee R.E.C., Faeder J.R. (2017). Demystifying the cytokine network: Mathematical models point the way. Cytokine.

[21] Thomas S., Bonchev D. (2010). A survey of current software for network analysis in molecular biology. Hum. Genom..

[22] Van Riel N.A. (2006). Dynamic modelling and analysis of biochemical networks: Mechanism-based models and model-based experiments. Brief. Bioinf..

[23] Scardoni G., Laudanna C., Zhang Y. (2012). Centralities based analysis of complex networks. New Frontiers in Graph Theory.

[24] Abedi M., Gheisari Y. (2015). Nodes with high centrality in protein interaction networks are responsible for driving signaling pathways in diabetic nephropathy. PeerJ.

[25] Peng Q., Schork N.J. (2014). Utility of network integrity methods in therapeutic target identification. Front. Genet..

[26] DeBoer J., Jagadish T., Haverland N.A., Madson C.J., Ciborowski P., Belshan M. (2014). Alterations in the nuclear proteome of HIV-1 infected T-cells. Virology.

[27] Parvizi J., Tan T.L., Goswami K., Higuera C., Della Valle C., Chen A.F., Shohat N. (2018). The 2018 definition of periprosthetic hip and knee infection: An evidence-based and validated criteria. J. Arthroplast..

[28] Qasim S.N., Swann A., Ashford R. (2017). The DAIR (debridement, antibiotics and implant retention) procedure for infected total knee replacement—A literature review. Sicot J..

[29] Hulse R.E., Kunkler P.E., Fedynyshyn J.P., Kraig R.P. (2004). Optimization of multiplexed bead-based cytokine immunoassays for rat serum and brain tissue. J. Neurosci. Methods.

[30] Couture C., Desjardins P., Zaniolo K., Germain L., Guérin S.L. (2018). Enhanced wound healing of tissue-engineered human corneas through altered phosphorylation of the CREB and AKT signal transduction pathways. Acta Biomater..

[31] Atalay M., Oksala N., Lappalainen J., Laaksonen D.E., Sen C.K., Roy S. (2009). Heat shock proteins in diabetes and wound healing. Curr. Protein Pept. Sci..

[32] Reid M.B., Li Y.P. (2001). Cytokines and oxidative signalling in skeletal muscle. Acta Physiol. Scand..

[33] Yang M., Guan D.W., Xiong C.Y., Cheng Z.H., Yu T.S. (2009). Expression of c-jun during the incised wound healing in mice skin. Fa Yi Xue Za Zhi.

[34] Hosokawa R., Urata M.M., Ito Y., Bringas P., Chai Y. (2005). Functional significance of Smad2 in regulating basal keratinocyte migration during wound healing. J. Investig. Dermatol..

[35] Nédellec S., Renaudineau Y., Bordron A., Berthou C., Porakishvili N., Lydyard P.M., Pers J.O., Youinou P. (2005). B cell response to surface IgM cross-linking identifies different prognostic groups of B-chronic lymphocytic leukemia patients. J. Immunol..

[36] Vollmar B., El-Gibaly A.M., Scheuer C., Strik M.W., Bruch H.P., Menger M.D. (2002). Acceleration of cutaneous wound healing by transient p53 inhibition. Lab. Invest..

[37] Matsubayashi Y., Ebisuya M., Honjoh S., Nishida E. (2004). ERK activation propagates in epithelial cell sheets and regulates their migration during wound healing. Curr. Biol..

[38] Zhao M. (2007). PTEN: A promising pharmacological target to enhance epithelial wound healing. Br. J. Pharmacol..

[39] Downward J. (1999). How BAD phosphorylation is good for survival. Nat. Cell Biol.

[40] Lin L., White S.A., Hu K. (2019). Role of p90RSK in kidney and other diseases. Int. J. Mol. Sci..

[41] Bao P., Kodra A., Tomic-Canic M., Golinko M.S., Ehrlich H.P., Brem H. (2009). The role of vascular endothelial growth factor in wound healing. J. Surg. Res..

[42] Zhang J., Bárdos T., Shao Q., Tschopp J., Mikecz K., Glant T.T., Finnegan A. (2003). IL-4 potentiates activated T cell apoptosis via an IL-2-dependent mechanism. J. Immunol..

[43] Matias M.A., Saunus J.M., Ivanovski S., Walsh L.J., Farah C.S. (2011). Accelerated wound healing phenotype in Interleukin 12/23 deficient mice. J. Inflamm..

[44] Kaviratne M., Hesse M., Leusink M., Cheever A.W., Davies S.J., McKerrow J.H., Wakefield L.M., Letterio J.J., Wynn T.A. (2004). IL-13 activates a mechanism of tissue fibrosis that is completely TGF-beta independent. J. Immunol..

[45] Li A., Feng M., Li Y., Liu Z. (2016). Application of outlier mining in insider identification based on boxplot method. Proc. Comput. Sci..

[46] Bland J.M., Altman D.G. (1995). Multiple significance tests: The Bonferroni method. BMJ.

[47] Ghosh S., Zang S., Mitra P.S., Ghimbovschi S., Hoffman E.P., Dutta S.K. (2011). Global gene expression and ingenuity biological functions analysis on PCBs 153 and 138 induced human PBMC in vitro reveals differential mode(s) of action in developing toxicities. Environ. Int..

[48] Bonnet A., Lagarrigue S., Liaubet L., Robert-Granié C., Sancristobal M., Tosser-Klopp G. (2009). Pathway results from the chicken data set using GOTM, Pathway Studio and Ingenuity softwares. BMC Proc..

[49] Han A.A., Currie H.N., Loos M.S., Vrana J.A., Fabyanic E.B., Prediger M.S., Boyd J.W. (2016). Spatiotemporal phosphoprotein distribution and associated cytokine response of a traumatic injury. Cytokine.

[50] Monastero R.N., Pentyala S. (2017). Cytokines as biomarkers and their respective clinical cutoff levels. Int. J. Inflam..

[51] Gawel D.R., Serra-Musach J., Lilja S., Aagesen J., Arenas A., Asking B., Bengnér M., Björkander J., Biggs S., Ernerudh J. (2019). A validated single-cell-based strategy to identify diagnostic and therapeutic targets in complex diseases. Genome Med..

[52] Heim C.E., Vidlak D., Scherr T.D., Hartman C.W., Garvin K.L., Kielian T. (2015). IL-12 promotes myeloid-derived suppressor cell recruitment and bacterial persistence during Staphylococcus aureus orthopedic implant infection. J. Immunol..

[53] Anderson J.M., McNally A.K. (2011). Biocompatibility of implants: Lymphocyte/macrophage interactions. Semin. Immunopathol..

[54] Peranteau W.H., Zhang L., Muvarak N., Badillo A.T., Radu A., Zoltick P.W., Liechty K.W. (2008). IL-10 overexpression decreases inflammatory mediators and promotes regenerative healing in an adult model of scar formation. J. Investig. Dermatol..

[55] Han A.A., Currie H.N., Loos M.S., Scardoni G., Miller J.V., Prince N., Mouch J.A., Boyd J.W. (2018). The impact of cytokine responses in the intra- and extracellular signaling network of a traumatic injury. Cytokine.

[56] Lee S., Kim M.S., Jung S.J., Kim D., Park H.J., Cho D. (2018). ERK activating peptide, AES16-2M promotes wound healing through accelerating migration of keratinocytes. Sci. Rep..

[57] François F., Godinho M.J., Grimes M.L. (2000). CREB is cleaved by caspases during neural cell apoptosis. FEBS Lett..

[58] Mayer C., Franz A., Harmsen J.F., Queitsch F., Behringer M., Beckmann J., Krauspe R., Zilkens C. (2017). Soft-tissue damage during total knee arthroplasty: Focus on tourniquet-induced metabolic and ionic muscle impairment. J. Orthop..

[59] Kamal A.H.M., Fessler M.B., Chowdhury S.M. (2018). Comparative and network-based proteomic analysis of low dose ethanol- and lipopolysaccharide-induced macrophages. PLoS ONE.

